# Advance in the Role of Epigenetic Reprogramming in Somatic Cell Nuclear Transfer-Mediated Embryonic Development

**DOI:** 10.1155/2021/6681337

**Published:** 2021-02-04

**Authors:** Xiaolei Zhang, Shaorong Gao, Xiaoyu Liu

**Affiliations:** ^1^Institute for Regenerative Medicine, Shanghai East Hospital, Shanghai Key Laboratory of Signaling and Disease Research, Frontier Science Center for Stem Cell Research, School of Life Sciences and Technology, Tongji University, Shanghai 200092, China; ^2^Clinical and Translational Research Center of Shanghai First Maternity and Infant Hospital, Shanghai Key Laboratory of Signaling and Disease Research, Frontier Science Center for Stem Cell Research, School of Life Sciences and Technology, Tongji University, Shanghai 200092, China; ^3^Tsingtao Advanced Research Institute, Tongji University, Qingdao 266071, China

## Abstract

Somatic cell nuclear transfer (SCNT) enables terminally differentiated somatic cells to gain totipotency. Many species are successfully cloned up to date, including nonhuman primate. With this technology, not only the protection of endangered animals but also human therapeutics is going to be a reality. However, the low efficiency of the SCNT-mediated reprogramming and the defects of extraembryonic tissues as well as abnormalities of cloned individuals limit the application of reproductive cloning on animals. Also, due to the scarcity of human oocytes, low efficiency of blastocyst development and embryonic stem cell line derivation from nuclear transfer embryo (ntESCs), it is far away from the application of this technology on human therapeutics to date. In recent years, multiple epigenetic barriers are reported, which gives us clues to improve reprogramming efficiency. Here, we reviewed the reprogramming process and reprogramming defects of several important epigenetic marks and highlighted epigenetic barriers that may lead to the aberrant reprogramming. Finally, we give our insights into improving the efficiency and quality of SCNT-mediated reprogramming.

## 1. Introduction

Somatic cell nuclear transfer (SCNT), first demonstrated by Gurdon in 1962 [1], is a technology to form reconstructed embryos by injecting donor nucleus into enucleated oocytes and generate cloned animals. The success of SCNT makes the transition from terminally differentiated cells to totipotent cells a reality [2]. It has been about two decades that the first cloned mammal, “Dolly,” the sheep, was born [3]. Since then, investigations on SCNT and cloned animals boomed, and different species were successfully cloned by various donor cell types [4–6]. In 2018, the first nonhuman primate species has been cloned by using fetal fibroblasts as donor cells [7]. Besides animal cloning, SCNT technology is widely used to acquire nuclear transfer embryonic stem cells (ntESCs), which is called therapeutic cloning [8–10]. The derivation of human ntESCs, which was first achieved at 2013 [11] and further improved in the following years [12–14], implies SCNT technology holds great application prospects in human therapeutics.

Although successful, low efficiency (Table 1) as well as defects in extraembryonic tissues and cloned individuals in many species impedes the application of SCNT technology, which has been fully reviewed [4, 15, 16]. SCNT embryos are often arrested at the early stages of preimplantation development. For the most used animal model, mouse, SCNT embryos are usually arrested at 2-cell and 4-cell stages [17, 18]. Even if the embryos develop to blastocyst stage, postimplantation defects and abnormal placentas, like enlarged placenta, were still observed [19]. Only about 1-2% of reconstructed embryos enable to develop to term [4, 20]. For other species, the highest cloning efficiency was demonstrated in bovine, which is about 5-20%, still much lower than that of IVF (about 40-60%) [20]. Even after born, abnormalities may still exist, for example, large offspring syndrome, failure of the immune system, and respiratory disorders [19, 20]. Although abnormal phenotypes exist, cloned animals are mostly fertile and the offspring show normal phenotypes [21–23]. Therefore, the abnormalities are largely caused by epigenetic reprogramming defects rather than genetic mutations. Indeed, it has been reported that aberrant reprogramming and epigenetic memories inherited from donor cells are barriers that impede reprogramming [17, 18, 24–27]. Therefore, understanding of epigenetic reprogramming process is essential for prompting the improvement of SCNT technology.

Up to date, great efforts have been made to improve cloning efficiency. However, due to the limitation of methodology and the scarcity of the required materials, especially 1-cell and 2-cell stage embryos, the progress went slowly. While with the development and improvement of low input high throughput sequencing technology, higher resolution of genome-wide epigenetic modification landscapes in SCNT embryos were detected, and our understanding of epigenetic reprogramming becomes clearer [18, 24, 26, 28, 29].

In this review, we will summarize our current knowledge on epigenetic reprogramming, mainly on DNA methylation, histone modifications, histone variants, X chromosome inactivation (XCI), chromatin accessibility, and 3D chromatin structures during SCNT embryo development and recent progress on elevating cloning efficiency and quality. Focusing on how to overcome reprogramming barriers to facilitate SCNT reprogramming and further improve reproductive as well as therapeutic cloning.

## 2. DNA Methylation

DNA methylation (5-methylcytosine, 5mC) is an epigenetic mark that occurs at cytosine residues in the CpG dinucleotide, generally regarded as associated with transcriptional silencing [30]. About 60-80% of the CpG sites in the mammalian genome are modified by 5mC [31]. DNMT3A and DNMT3B are two methyltransferases essential for *de novo* DNA methylation, and DNMT1 is responsible for its maintenance during embryogenesis [32–34]. DNA demethylation is triggered by ten-eleven translocation (TET) protein-mediated oxidation from 5mC to 5-hydroxymethylcytosine (5hmC) followed by thymine DNA glycosylase- (TDG-) mediated base excision repair [35–37]. In mouse, both maternal and paternal alleles undergo demethylation through active and/or passive manner after fertilization and finally reached the lowest level at the blastocyst stage [38, 39]. It has been reported that knockout of *Dnmt3a* and *Dnmt3b* leads to mouse infertility [32, 40], and deletion of *Tet3* causes an increased frequency of developmental failure in embryos [41], suggesting that optimized DNA methylation pattern is essential for normal development. Thus, a DNA methylation pattern that resembled that of fertilized embryos may be a permissive state for SCNT embryo development.

### 2.1. DNA Methylation Is Globally Reprogrammed during SCNT Embryo Development

Given that somatic donor cells usually possess high DNA methylation levels [31], SCNT embryos must undergo global demethylation to reprogram the DNA methylation pattern of somatic cells to that of fertilized embryos. After activation, oocyte-stored TET3 immediately incorporated into pseudopronucleus (PPN) of the reconstructed embryo to catalyze conversion from 5mC to 5hmC, which implies active demethylation during SCNT embryo development [42], bearing resemblance with normal embryo development [42, 43]. Whole-genome bisulfite sequencing (WGBS) of mouse SCNT blastocysts revealed a very low DNA methylation level (15.6%) similar to that of IVF blastocysts (19.1%) [25]. Considering the high methylation level of the donor mouse embryonic fibroblast (MEF) cells (78%) used in the study, the result indicates successful global reprogramming of DNA methylation state. But this demethylation has not completed when the mouse SCNT embryos developed to the late 1 cell stage [28]. Our lab analyzed DNA methylation levels of SCNT embryos by using an embryo biopsy system along with single-cell reduced representation bisulfite sequencing (RRBS), and the results showed that at 2- and 4-cell stage, the SCNT samples possessed generally higher methylation level than the corresponding fertilized embryos [18], suggesting global demethylation in SCNT embryos may require several rounds of replication delusion.

### 2.2. Aberrant DNA Methylation Reprogramming in SCNT Embryos

Although successful global demethylation in blastocyst, aberrant DNA methylation patterns can be detected in SCNT embryos, even after implantation [5, 44, 45]. In mouse 4-cell stage SCNT embryos, especially arrested samples, the averaged methylation levels on gene body regions were significantly increased, resembling the trend of donor cells [18]. Similarly, cloned, but not fertilized, bovine morula possesses highly methylated nuclei in all blastomeres that resembled those of the fibroblast donor cells [44].

**Figure 1:**
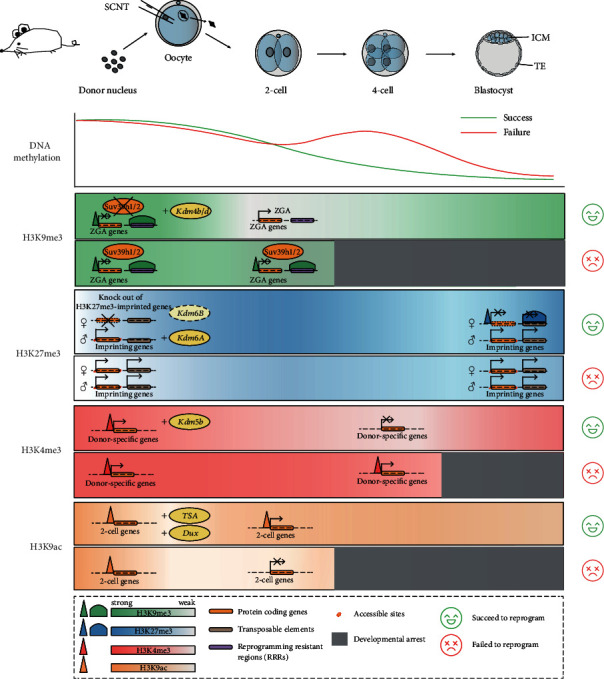
Epigenetic reprogramming of DNA methylation and histone modifications during mouse SCNT embryo development. DNA methylation: somatic donor cells usually possess high DNA methylation levels. After activation, the reconstructed embryos undergo global DNA demethylation although this demethylation has not been completed at the late 1 cell stage of SCNT embryos and requires several rounds of replication delusion. However, there is an aberrant remethylation in arrested 4 cell stage SCNT embryos and reduction of the inappropriate DNA methylation rescued the developmental arrest. *H3K9me3*: during SCNT embryo development, some zygotic genome activation (ZGA) genes and reprogramming resistant regions (RRRs) harbor donor cell-inherited H3K9me3 mark, which may be the cause of reprogramming failure. Removal of donor-inherited H3K9me3 either by ectopic expressing *Kdm4b/d* (H3K9me3-specific demethylases) or knockdown of *Suv39h1/2* (H3K9 methyltransferases) can help the embryo overcoming the reprogramming defects. *H3K27me3*: loss of H3K27me3-mediated imprinting leads to defects of extraembryonic tissues of SCNT embryos, such as large placenta phenotype. Although overexpression of H3K27me3-specific demethylase KDM6A elevated blastocyst developmental rate but not full-term development, both knock out of H3K27me3-imprinted genes and knockdown KDM6B can help SCNT embryos undergo successful reprogramming. *H3K4me3*: donor-inherited H3K4me3 is defined as an epigenetic barrier of SCNT reprogramming. H3K4me3 demethylation by *Kdm5b* overexpression is an important step to overcome reprogramming failure. *H3K9ac*: during SCNT development, aberrant H3K9ac regions impair ZGA. TSA treatment and Dux overexpression can correct the aberrant H3K9ac signal and help the embryos achieve successful reprogramming.

RRBS on 1 cell stage mouse SCNT embryos uncovered more than 20 genes, along with long interspersed elements (LINEs) and long terminal repeats (LTRs) defined as demethylation-resistant regions [28]. Nevertheless, by using ultralow-input WGBS, Gao et al. found that the persistently methylated differentially methylated regions (pDMRs) were moderately similar in arrest and normally developed NT embryos and were more frequently inherited from cleaved embryos to blastocyst stage, which reflects their functional irrelevance in the arrest of SCNT reprogramming [24]. Furthermore, they identified wide-spread regions that were aberrantly remethylated in SCNT embryos compared to the IVF counterparts, called remethylated differentially methylated regions (rDMRs), which are twice in arrested samples as many as in normally developed NT embryos (Figure 1). These rDMRs lead to misexpression of genes and retrotransposons important for zygotic genome activation (ZGA). Reduction of inappropriate DNA methylation rescued the developmental arrest at cleavage stages and facilitated proceeding to blastocyst development, increasing the blastocyst rate to 48.2% (compared to control of 39.5%) [24]. In conclusion, excessive DNA remethylation is a potent barrier that limits the full-term development of SCNT embryos, but the role of somatic-inherited DNA methylation still needs further proven, after all, an optimized DNA methylation pattern that resembled that of fertilized embryos is essential for SCNT reprogramming.

## 3. Histone Modifications

In eukaryotic cells, the basic functional unit of chromatin is the nucleosome, containing ~147 bp genomic DNA wrapped around a core histone octamer. Covalent histone modifications, such as acetylation, methylation, ubiquitination, and phosphorylation, are major epigenetic marks that regulate transcription [46–48]. Successful reprogramming of SCNT embryos should include reprogramming of histone modification patterns from somatic donor cells to those of normal embryos. Here, we will discuss the roles of several major histone modifications, including trimethylation at the 9^th^ lysine residue of histone H3 (H3K9me3), trimethylation at the 27^th^ lysine residue of histone H3 (H3K27me3), trimethylation at the 4^th^ lysine residue of histone H3 (H3K4me3), and histone acetylation on SCNT reprogramming.

### 3.1. Aberrant H3K9me3 Reprogramming Impairs Preimplantation Development

H3K9me3 has been shown to play important roles in heterochromatin formation and repression of gene expression in various types of cells, including preimplantation embryos [7, 49]. In 2014, Matoba and colleagues identified 222 reprogramming resistant regions (RRRs) that failed to be activated in SCNT 2-cell embryos compared to IVF 2-cell embryos. Interestingly, these RRRs are enriched for H3K9me3 in somatic cells [17]. Removal of this epigenetic mark either through ectopic expression of *Kdm4d* (an H3K9me3-specific demethylase) in oocytes or knockdown of *Suv39h1* and *Suv39h2* (two H3K9 methyltransferases) in donor MEF cells not only attenuated the ZGA defect but also improved the reprogramming efficiency of SCNT embryos [17]. Further investigations by Liu et al. identified 7248 genes resisted donor-liked H3K9me3 signal at promoters in 2-cell stage SCNT embryos. Removal of the H3K9me3 mark inherited from donor cells by injecting *Kdm4b* helped the SCNT embryos go over 2-cell arrest and finally significantly elevated the potential of ntESC derivation, blastocyst rate, and even birth rate [18] (Figure 1). In bovine, KDM4D and KDM4E function as regulators that help SCNT embryos to break through H3K9me3 barriers [50]. Moreover, the expression of H3K9me3 demethylase Kdm4d/4a could reduce H3K9me3 level and significantly improve the efficiency of human SCNT blastocyst and ntESC cell line formation [13]. And the use of *Kdm4d* combined with histone deacetylase inhibitor (HDACi) trichostatin A (TSA) treatment successfully generated cloned cynomolgus (*Macaca fascicularis*) monkeys by using adult cumulus cells as donor cells [7], although the positive effect of TSA treatment might be functionally linked to H3K9me3 removal in mouse due to unchanged development potential by TSA treatment with *Kdm4d*-mRNA-injected mouse SCNT embryos [17]. The results above imply a conserved barrier of H3K9me3 inherited from donor cells during SCNT reprogramming in mammalian species.

Although the use of *Kdm4d* in SCNT results in an implantation rate comparable with that of IVF, only less than 15% of the implanted SCNT embryos develop to term, and abnormal large placentae are still observed in *Kdm4d*-injected SCNT embryos [17]. Additionally, Kdm4A addition was not able to enhance the in vivo long-term development capacity of porcine SCNT embryo [51], indicating H3K9me3 may mainly impede preimplantation development of SCNT embryos and other barriers may affect postimplantation development.

### 3.2. H3K27me3 Reprogramming Defects Are Obstacles in Pre- and Postimplantation SCNT Embryos

H3K27me3 is an epigenetic regulator widely known as a transcription repressor [52, 53]. During mouse preimplantation development, H3K27me3 is rapidly lost at both maternal and paternal alleles followed by dynamic especially when lineage specification of inner cell mass (ICM) and trophectoderm (TE) [54, 55]. Lots of studies have elucidated the critical role of H3K27me3 during both pre- and postimplantation embryo development [54, 56–59].

Aberrant H3K27me3 reprogramming may be a barrier of SCNT embryo development in various species [25, 60, 61]. Okae et al. identified three DNA methylation-independent imprinted genes *Gab1*, *Sfmbt2*, and *Slc38a4* showed loss of imprinting in all cloned mouse embryos [62], which might be involved in placentomegaly of cloned mouse when considering their important roles in placental development [63, 64]. Further studies found 76 genes with paternal allele-specific DNase I hypersensitive sites (DHSs) that are devoid of DNA methylation but harbor maternal allele-specific H3K27me3 [65]. Interestingly, all the three genes above are included in the 76 genes, which rise the suspect that the defect of H3K27me3 mediated imprinting may cause the abnormality of SCNT placentae. Indeed, many groups proved that loss of H3K27me3-imprinting in SCNT embryos disrupts mouse postimplantation development, and this defect can be detected earliest in blastocyst stage embryos up to now [25, 66, 67]. However, whether this defect exists more earlier in SCNT embryos requires further exploration [68]. A recent study found that the majority of H3K27me3-mediated imprinting regions are located to solo ERVK LTR repeats, which act as imprinted transcription initiation sites for noncoding RNAs and chimeric mRNA in extraembryonic tissues [69]. It is possible that the defects of H3K27me3 reprogramming are relevant to aberrant expression of transposable element during SCNT embryo development. Although restore the normal paternal expression of H3K27me3-imprinting genes (*Sfmbt2*, *Gab1*, and *Slc38a4*) in SCNT placentae by maternal knockout unchanged the enlarged placentae state [66], both correcting the expression of clustered miRNAs within the *Sfmbt2* gene and quadruple monoallelic deletion of *Sfmbt2*, *Jade1*, *Gab1*, and *Smoc1* ameliorates the placental phenotype, especially *Sfmbt2* [66, 67].

Apart from the impact of loss of H3K27me3-imprinting on SCNT postimplantation, another group demonstrated H3K27me3 as an obstacle of SCNT preimplantation development. Overexpression of the H3K27me3-specific demethylase KDM6A significantly increased the SCNT blastocyst formation rate but did not improve the rate of full-term development, implies lack of KDM6A may be not the reason for loss of H3K27me3-dependent imprinting, at least in mouse. Contrastingly, knockdown of KDM6B not only facilitated ZGA and improved the blastocyst formation rate but also increased birth rate and ntESC establishment efficiency [68] (Figure 1). Collectively, both deposition on specific regions (like H3K27me3-imprinting genes) and appropriate removal of H3K27me3 are important for successful SCNT reprogramming although underlaid mechanisms are still unknown.

### 3.3. Somatic Inherited H3K4me3 Is a Potent Barrier of SCNT-Mediated Reprogramming

H3K4me3 is usually associated with transcriptional activation. Many groups have depicted the pattern of H3K4me3 during preimplantation in mouse [54, 70, 71]. Both appropriate removal of noncanonical H3K4me3 by *Kdm5b* in oocyte and establishment of canonical and broad H3K4me3 in preimplantation embryos are essential for normal mouse development [54, 70]. Unlike the well-described H3K4me3 pattern in normal mouse preimplantation embryos, studies about the whole H3K4me3 pattern during SCNT reprogramming have not been reported until now.

In 2016, we found that *Kdm5b* failed to be activated in 4-cell-arrest SCNT embryos. Injection of si-*Kdm5b* in MII oocytes largely reduced the rate of high-quality blastocyst development, and overexpression of *Kdm5b* helped the SCNT embryos to pass 4-cell arrest and significantly increased blastocyst formation rate and quality. What is more, the gene expression levels of NT 4-cell embryos were largely rescued by the overexpression of *Kdm5b* [18]. Considering the role of *Kdm5b* as H3K4me3 demethylase and the function of H3K4me3 on transcription initiation, it is possible that H3K4me3 mark with donor-specific signature may be a barrier of SCNT reprogramming (Figure 1). This point has been proved in *Xenopus*, human, and bovine SCNT embryos that donor-inherited H3K4me3 acts as an epigenetic barrier impacts SCNT reprogramming [72, 73]. H3K4me3 demethylation by *Kdm5b* overexpression not only attenuated ON-memory genes (genes highly expressed in donor cells and SCNT embryos but not IVF embryos) but also improved cloning efficiency. The results indicate that removal of the donor-specific H3K4me3 mark may efficiently reprogram the SCNT embryos but much more further investigations about roles of H3K4me3 during SCNT-mediated reprogramming need to be performed.

### 3.4. Aberrant Histone Acetylation Impairs the SCNT Efficiency

Histone acetylation usually occurs on the lysine residues of core histones and marks both promoters and enhancers. Acetylation has the potential to loosen nucleosome configuration and increase chromatin accessibility for transcription factors [74]. During ZGA, the persistent accessible enhancers are marked by H3K27ac and characterized by distal H3K4me3 deposition in human early embryos, while the poised enhancers are likely to be activated in later development by remarked H3K27ac in a tissue-specific manner [75]. In early zebrafish embryos, widespread H3K27ac deposition is found to be required for gene activation [76]. This indicates that histone acetylation reprogramming is another critical step for early embryo development.

When somatic cell nuclei are injected into the enucleated MII oocytes, the acetylated lysine residues are quickly deacetylated and then reacetylated after activation. The reestablishment of histone acetylation is essential for zygotic gene activation in cloned embryos [77]. However, several acetylation marks on histones, such as H4K8ac and H4K12ac, are persisted in the genome during SCNT, which may be responsible for the low cloning efficiency. On the other hand, histone deacetylase inhibitors (HDACi), which can improve histone acetylation and the success rate of cloning significantly, have been widely used during SCNT [78]. Recently, our group generated the genome-wide H3K9ac map during SCNT development and found the aberrant acetylated regions impair the zygotic gene activation. TSA treatment and Dux overexpression can correct the aberrant H3K9ac signal [79] (Figure 1). These suggest the reestablishment of histone acetylation is also a necessary part of epigenetic reprogramming. It should be noted that HDACi treatment can also improve nascent mRNA production [80] and gene expression [81] during SCNT embryo development, so the mechanism of HDACi treatment improves cloning efficiency still deserve further investigation.

## 4. Histone Variants

Aside from the canonical histones, histone variants endow chromatin critical functions, and their roles in oocyte-mediated reprogramming have been reviewed elsewhere [82–85]. The mammalian sperm genome is packaged into highly condensed chromatin consisting primarily of protamine but 5-15% residual histones. After fertilization, the paternal genome undergoes dramatic chromatin remodeling, and maternally stored histones, such as H3.3 (coded by *H3f3a* and *H3f3b*), are incorporated into the sperm nucleus as early as 1 h after fertilization [85]. And the incorporation is essential for the activation of the paternal genome and preimplantation development during embryogenesis [86].

Although the somatic cell genome is packaged by histones rather than protamine, global chromatin remodeling was still observed [85, 87]. After activation, donor cell-derived histone H3 variants H3.1, H3.2, and H3.3, as well as H2A, H2A.Z, and microH2A, were rapidly eliminated from the chromatin [87, 88]. All the three oocyte-stored H3 variants, H2A.X, and oocyte-specific H1 variant, H1FOO, were incorporated into the donor genome within minutes of nuclear transfer [87, 89, 90]. Knockdown of histone variant H3.3 in mouse oocytes results in compromised reprogramming and downregulation of key pluripotent genes, and this compromised reprogramming was rescued by injecting exogenous H3.3 mRNA, but not H3.2 mRNA into oocytes [85], revealing a critical role of optimized chromatin variants incorporation in normal SCNT reprogramming.

## 5. X Chromosome Inactivation (XCI)

XCI is a remarkable event during normal embryogenesis [62, 91]. X chromosome is inactivated during spermatogenesis. During mouse embryogenesis, the paternal X chromosome is reactivated at the 2-cell stage. After that, the paternal X chromosome will be silenced again through an imprinted manner and persisted in extraembryonic lineages. In contrast, the paternal X chromosome is reactivated in the epiblast in the late blastocyst, then, the X chromosome from maternal or paternal genomes is randomly inactivated during embryo development [92–94]. The precise regulation of dynamic activity of the X chromosome is crucial for the epigenetic reprogramming during early embryo development [95].

XCI ensures a similar dosage of X-linked genes between male and female cells. However, this event in SCNT embryos is largely abnormal among species [27, 51]. In mouse SCNT embryos, X-linked genes were largely downregulated, which is caused by ectopic expression of *Xist* from the active X chromosome regardless of sex, leading to abnormal inactivation of both X chromosomes [27]. Similarly, *Xist* is also known to be aberrantly expressed in bovine and pig SCNT embryos and proven to be associated with prenatal death [96, 97], suggesting excessive *Xist* expression may be a barrier of SCNT-mediated reprogramming. Deletion of *XIST* on the active X chromosome rescued global gene expression and resulted in about an 8- to 9-fold increase in cloning efficiency [27]. Concordantly, prior injection of *Xist*-siRNA into reconstructed oocytes normalized global gene expression of mouse SCNT embryos at the morula stage and further improved cloning efficiency 10-folds higher than control [98]. Moreover, correction of the abnormal XCI has a synergistic effect with TSA but ectopic activation of *Xist* is reprogramming barrier independent of H3K9me3 inherited from donor cells [25, 98]. Differently in pig, abnormal XCI seems linked with H3K9me3 for that increased quality of *XIST*-deficient SCNT embryos was associated with the global H3K9me3 reduction and vice versa; *Kdm4a* addition also induced *XIST* derepression in the active X chromosome [51]. This discrepancy may be a result of different XCI processes among different species, and the underlaid mechanisms require further understanding.

## 6. Chromatin Accessibility

Chromatin accessibility is a good indicator of transcriptional regulatory elements and can serve as a predictor of gene transcription activity. In recent years, with the development and improvement of low-input DNase I hypersensitive sequencing (liDNase-seq) and assay for transposase-accessible chromatin using sequencing (ATAC-seq), accessible chromatin sites of mouse and human preimplantation embryos enabled to be profiled [29, 65, 99, 100]. By using liDNase-seq, Lu et al. uncovered that DNase I-hypersensitive site (DHS) landscape is progressively established with a drastic increase at the 8-cell stage of mouse preimplantation embryos [29]. The global chromatin de- and recondensation is likely promoted by cis-regulating of LINE-1 transcriptional activity [101]. Transcription factors Nfya and Oct4 were responsible for DHS formation at 2- and 8-cell stage embryos, respectively [29].

Full-pattern of chromatin accessibility during mouse and human SCNT embryo development has not been elucidated, but a recent research profiled DHSs in donor cells and late-1-cell stage mouse SCNT embryos. They found SCNT-mediated reprogramming of chromatin accessibility is largely completed by 12 h after activation because DHSs of the donor cells are drastically changed to recapitulate that of the IVF zygotes within 12 h. Surprisingly, this change is DNA replication-independent, which is conserved in *Xenopus* SCNT embryos [102], and the switch from donor-specific TF network to that of zygotic may be the critical factor responsible for the DHS profile reprogramming [103].

Despite global reprogramming, some regions are resistant to reprogram [103]. Failure to close accessible somatic promoters or to open distal regulatory regions required for differentiation program may be the major reprogramming barriers. It is interesting that these regions are enriched for H3K9me3, a robust reprogramming barrier discussed above, in both donor cells and 2-cell SCNT embryos [103]. Considering the change of the TF network which accompanies with this reprogramming, failure of specific somatic cell TFs to dissociate from chromatin can also be a barrier in SCNT reprogramming. ATAC-seq on *Xenopus* SCNT embryos revealed great loss of chromatin accessible sites before first cleavage compared to that of donor cells, which is concordant with the pattern in mouse. The researchers found genes that are silenced but have preexisting open transcription start sites (TSSs) in donor cells are prone to be activated after SCNT, while genes resistant to reprogramming are associated with closed chromatin configurations [102]. It is possible that preexisted open accessibility of donor-specific genes and closed accessibility of zygotic-essential genes inherited from donor cells may be barriers during SCNT reprogramming, but it needs further proven.

## 7. Higher-Order Chromatin Structure

Chromatin in the nucleus of eukaryotic cells is packaged in a hierarchical structure, which is associated with many biological processes [104, 105]. The role of the 3D genome organization during mammalian embryogenesis has been investigated benefit from the advance of the low-input Hi-C (genome-wide chromosome conformation capture) technology in recent years [106–108], which reveals the removal and reestablishment of chromatin higher-order structure are essential for both mouse [106, 107] and human [108] embryogenesis.

**Figure 2:**
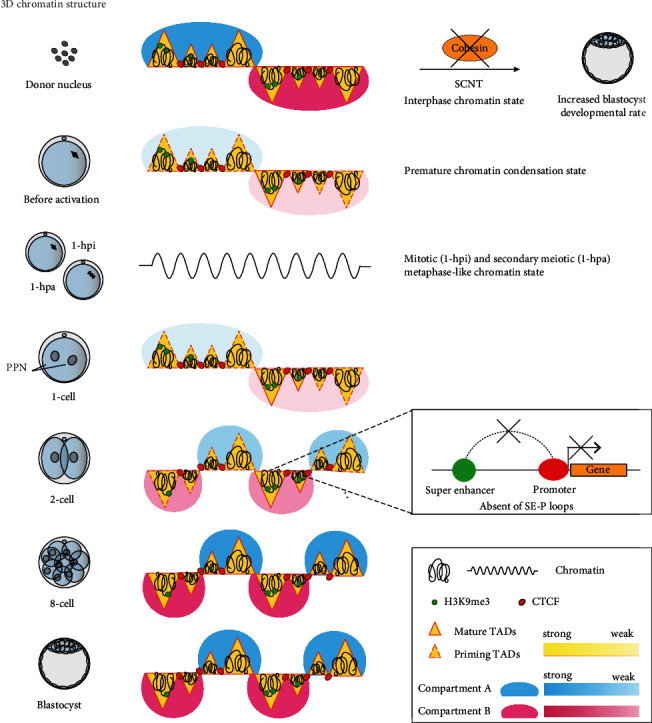
The higher-order chromatin organization in mouse SCNT embryos. Somatic donor cells exhibit interphase-state chromatin characterized by mature compartments and topologically associating domains (TADs). Before activated, the transferred nucleus first enters a mitotic-like state (premature chromatin condensation) followed by exhibiting mitotic and secondary meiotic metaphase-like chromatin states lacking compartments and TADs 1 hour postinjection (1-hpi) and 1-hour postactivation (1-hpa), respectively. TADs are stronger in SCNT 1-cell stage embryos and then become weaker at the 2-cell stage and gradually consolidating. Super enhancer-promoter (SE-P) loops that exist in fertilized 2-cell embryos are absent in SCNT 2-cell embryos, which is correlated with aberrant H3K9me3 and TAD persistence. Compartments A/B are markedly weak in 1-cell SCNT embryos and become increasingly strengthened afterward. By the 8-cell stage, somatic chromatin architecture is largely reset to embryonic patterns until the blastocyst stage. Predepleting cohesin in donor cells increases SCNT reprogramming efficiency.

A recent study of our group profiles the spatiotemporal dynamic of 3D chromatin structure in SCNT early embryos and reveals 3D chromatin structure can be rapidly reorganized to an embryo-like state after nuclear transfer. However, the aberrant TADs and compartment A/B organization can be observed and remain throughout preimplantation SCNT embryo development. Overexpression of KDM4B, a H3K9me3 demethylase, can partially improve the abnormal 3D chromatin structures [26] (Figure 2). This indicates a correlation between the organization of 3D chromatin structure and histone modifications during epigenetic reprogramming.

## 8. Removal of Multiple Barriers Is a Promising Approach to Improve SCNT Reprogramming

It has been over two decades that the first mammalian species has been successfully cloned, but low efficiency was still observed until recently. Numerous efforts have been made to increase reprogramming efficiency by removing epigenetic barriers. Matoba et al. found H3K9me3 inherited from donor cells act as a barrier that impede mouse SCNT-mediated preimplantation development. Removal of H3K9me3 in donor cells by injecting *Kdm4d* mRNA into reconstructed embryos 5 hours postactivation (hpa) significantly increased the blastocyst rate up to 81.2% (% blastocyst of cleaved embryos) regardless of donor cell types and elevated birth rate from only 1% up to 8.7%. Besides, the rate of ntESC line derivation was increased from 10.1% to 50% after *Kdm4d* injection. Moreover, to prevent the establishment of H3K9me3 in donor cells, they knock down *Suv39h1/2* (H3K9me3 transferases) in donor cells prior SCNT and improved blastocyst rate from 6.7% to 49.9% [17]. Accordantly, our lab found another H3K9me3 demethylase, *Kdm4b*, efficiently removed the H3K9me3 barrier to increase blastocyst rate from about 30% to over 80%. Simultaneously, we found H3K4me3 may be a candidate epigenetic barrier that impedes SCNT-mediated reprogramming. Injection of *Kdm5b* mRNA into enucleated oocyte significantly improves mouse blastocyst rate from about 30% to over 50%. It is worth noting that coinjection of *Kdm4b* and *Kdm5b* successfully elevated blastocyst rate over 95% and led to over 11% of cloned embryos developing to live animals, moreover, 60% ntESC derivation efficiency based on the total number of MII oocytes rise the possibility that removing multibarriers may be a more efficient way to improve cloning efficiency [18].

We found excessive remethylation is a potent epigenetic barrier in another study. Optimized DNA methylation level by injecting siRNAs of *Dnmt3a* and *Dnmt3b* into enucleated oocytes, 48.2% blastocysts were generated from cleaved embryos (39.5% blastocyst rate of control group). Furthermore, of enucleated oocytes that subjected to Kdm4b+5b mRNA and siDnmt3a+3b co-injection, 92.3% cleaved embryos developed to blastocyst stage [24]. Another study of Matoba et al. demonstrated that using a combination of Xist knockout donor cells and overexpression of Kdm4b, more than 20% birth rate of mouse SCNT embryos were achieved [25], which is coincided with the idea that removing multiple epigenetic barriers is a more efficient method for SCNT reprogramming.

## 9. Concluding Remarks

SCNT provides the only way to reprogram somatic cells into totipotent embryos and generate viable animals [9, 109, 110]. After injected into enucleated oocytes, the donor nucleus quickly undergoes nuclear membrane breakdown followed by premature chromosome condensation (PCC), which is triggered by the M-phase-prompting factors (MPFs) stored at ooplasm [111]. After activation, the nuclear membrane is reformed to envelop PPN and incorporates amounts of maternal factors [112]. Then, the reconstructed embryos undergo SCNT-mediated embryogenesis. However, only few of reconstructed embryos can develop to the blastocyst stage, let alone develop to term. ZGA failure and disrupted transcriptome were detected in SCNT embryos very often, and this is largely affected by aberrant epigenetic reprogramming [4].

In this review, we concluded our understanding on epigenetic barriers of SCNT-mediated reprogramming and methods to overcome these epigenetic berries. Given that removal of multiple barriers that impede SCNT-mediated reprogramming gives a blastocyst rate over 95% of cleaved embryos and ntESC derivation efficiency of 60% based on the total number of MII oocytes [18], and over 20% birth rate of mouse [25], we demonstrate removing multiple barriers may be a more efficient approach to achieve complete reprogramming compared to single barrier removal. However, low birth rate compared with IVF counterpart and large placentae were still observed. Therefore, further studies need to focus on exploring more about reprogramming barriers and emphasizing on removing multiple barriers to achieve nearly complete SCNT reprogramming.

## Figures and Tables

**Table 1:** Cloning efficiency of inner-species SCNT-mediated reprogramming.

Species	Donor cell type	Total oocytes	Reconstructed oocytes	Cleaved embryo/rate	Blastocyst number/rate of cleaved embryo	Transferred embryo number	Birth pups	Birth rate of cleaved embryo	Birth rate of transferred embryo	References
Sheep	Adult mammary epithelium	—	277	247	29	29	1	0.40%	3.45%	[3]
Cow	Fetal fibroblasts (transgenic)	—	276	—	33/-	28	4	—	14.29%	[113]
Cow	Oviductal cells	150	88	77	20/25.97%	4	3	3.90%	75%	[114]
Cow	Adult cumulus	99	37	31	18/58.06%	6	5	16.13%	83.33%	[114]
Mouse	Adult cumulus cells	—	136	45	—	45 (transferred with 2-cell embryos)	7 (two died at day 6-7)	15.56%	15.56%	[115]
Goat	Fetal fibroblasts (transgenic)	—	138	48	—	47 (transferred with cleaved embryos)	1	2.13%	2.08%	[116]
Pig	Fetal fibroblasts	210	188	110	—	110 (2- and 8-cell stage embryos were transferred)	1	0.91%	0.91%	[117]
Pig	Granulosa cells	245	74	—	—	72	5	—	6.94%	[118]
Rabbit	Adult transgenic cumulus cells	—	775	—	—	371 (transferred with 4-cell stage embryos)	6	—	1.62%	[119]
Cat (*Felis domesticus*)	Adult cumulus cells	—	—	—	—	3	1	—	33.3%	[120]
Mule	Fetal fibroblasts	120	113	—	—	113 (transferred at different days)	1	—	0.88%	[121]
Horse	Adult skin fibroblasts	—	841	753	22/2.92%	22	1	0.13%	4.55%	[122]
Rat	Fetal fibroblasts	—	—	129	—	129 (transferred with 2-cell stage embryos)	2	1.55%	1.55%	[123]
Dog	Adult skin fibroblasts	—	—	1095	—	1095 (transferred with cleaved embryos)	2	0.18%	0.18%	[124]
Ferret	Adult cumulus cells	—	487	—	—	375 (transferred immediately after activation)	2	—	0.53%	[125]
Buffalo	Fetal fibroblasts & adult granulosa cells	—	—	—	42/11.04-31.39%	42	5 (one died 20 min after birth & 1 died on day 14 after birth)	—	11.9%	[126]
Camel	Adult cumulus cells	75	58	—	-/(63.88 ± 8.66)	26	1	—	3.85%	[127]
Cynomolgus monkey	Fetal fibroblast	127	109	79	—	79 (transferred with 2-cell stage embryos)	2	2.53%	2.53%	[7]

## Abbreviations

SCNT: Somatic cell nuclear transfer
ntESCs: Nuclear transfer embryonic stem cells
XCI: X chromosome inactivation
5mC: 5-Methylcytosine
DNMT3A: DNA methyltransferase 3A
DNMT3B: DNA methyltransferase 3B
DNMT1: DNA methyltransferase 1
TET: Ten-eleven translocation
5hmC: 5-Hydroxymethylcytosine
TDG: Thymine DNA glycosylase
PPN: Pseudopronucleus
WGBS: Whole-genome bisulfite sequencing
MEF: Mouse embryonic fibroblast
RRBS: Reduced representation bisulfite sequencing
LINEs: Long interspersed elements
LTRs: Long terminal repeats
pDMRs: Persistently methylated differentially methylated regions
rDMRs: Remethylated differentially methylated regions
ZGA: Zygotic genome activation
H3K9me3: Trimethylation at the 9^th^ lysine residue of histone H3
H3K27me3: Trimethylation at the 27^th^ lysine residue of histone H3
H3K4me3: Trimethylation at the 4^th^ lysine residue of histone H3
HDACi: Histone deacetylase inhibitor
TSA: Trichostatin A
PRC2: Polycomb repressive complex 2
liDNase-seq: DNase I hypersensitive sequencing
ATAC-seq: Transposase-accessible chromatin using sequencing
DHS: DNase I-hypersensitive site
TSSs: Transcription start sites
hpa: Hour postactivation
PCC: Premature chromosome condensation
MPFs: M-phase-prompting factors.

## References

[1] Gurdon J. B. (1962). The developmental capacity of nuclei taken from intestinal epithelium cells of feeding tadpoles. *Journal of Embryology and Experimental Morphology*.

[2] Lu F., Zhang Y. (2015). Cell totipotency: molecular features, induction, and maintenance. *National Science Review*.

[3] Wilmut I., Schnieke A. E., McWhir J., Kind A. J., Campbell K. H. (1997). Viable offspring derived from fetal and adult mammalian cells. *Nature*.

[4] Matoba S., Zhang Y. (2018). Somatic cell nuclear transfer reprogramming: mechanisms and applications. *Cell Stem Cell*.

[5] Teperek M., Miyamoto K. (2013). Nuclear reprogramming of sperm and somatic nuclei in eggs and oocytes. *Reproductive medicine and biology*.

[6] Wang X., Qu J., Li J., He H., Liu Z., Huan Y. (2020). Epigenetic reprogramming during somatic cell nuclear transfer: recent progress and future directions. *Frontiers in Genetics*.

[7] Liu Z., Cai Y., Wang Y. (2018). Cloning of macaque monkeys by somatic cell nuclear transfer. *Cell*.

[8] Munsie M. J., Michalska A. E., O’Brien C. M., Trounson A. O., Pera M. F., Mountford P. S. (2000). Isolation of pluripotent embryonic stem cells from reprogrammed adult mouse somatic cell nuclei. *Current Biology*.

[9] Wakayama T., Tabar V., Rodriguez I., Perry A. C., Studer L., Mombaerts P. (2001). Differentiation of embryonic stem cell lines generated from adult somatic cells by nuclear transfer. *Science*.

[10] Byrne J. A., Pedersen D. A., Clepper L. L. (2007). Producing primate embryonic stem cells by somatic cell nuclear transfer. *Nature*.

[11] Tachibana M., Amato P., Sparman M. (2013). Human embryonic stem cells derived by somatic cell nuclear transfer. *Cell*.

[12] Chung Y. G., Eum J. H., Lee J. E. (2014). Human somatic cell nuclear transfer using adult cells. *Cell Stem Cell*.

[13] Chung Y. G., Matoba S., Liu Y. (2015). Histone demethylase expression enhances human somatic cell nuclear transfer efficiency and promotes derivation of pluripotent stem cells. *Cell Stem Cell*.

[14] Yamada M., Johannesson B., Sagi I. (2014). Human oocytes reprogram adult somatic nuclei of a type 1 diabetic to diploid pluripotent stem cells. *Nature*.

[15] Yang X., Smith S. L., Tian X. C., Lewin H. A., Renard J. P., Wakayama T. (2007). Nuclear reprogramming of cloned embryos and its implications for therapeutic cloning. *Nature Genetics*.

[16] Krishnakumar R., Blelloch R. H. (2013). Epigenetics of cellular reprogramming. *Current Opinion in Genetics & Development*.

[17] Matoba S., Liu Y., Lu F. (2014). Embryonic development following somatic cell nuclear transfer impeded by persisting histone methylation. *Cell*.

[18] Liu W., Liu X., Wang C. (2016). Identification of key factors conquering developmental arrest of somatic cell cloned embryos by combining embryo biopsy and single-cell sequencing. *Cell discovery*.

[19] Loi P., Iuso D., Czernik M., Ogura A. (2016). A new, dynamic era for somatic cell nuclear transfer?. *Trends in Biotechnology*.

[20] Ogura A., Inoue K., Wakayama T. (2013). Recent advancements in cloning by somatic cell nuclear transfer. *Philosophical transactions of the Royal Society of London. Series B, Biological sciences*.

[21] Tamashiro K. L., Wakayama T., Akutsu H. (2002). Cloned mice have an obese phenotype not transmitted to their offspring. *Nature Medicine*.

[22] Fulka J., Miyashita N., Nagai T., Ogura A. (2004). Do cloned mammals skip a reprogramming step?. *Nature Biotechnology*.

[23] Wakayama S., Kohda T., Obokata H. (2013). Successful serial recloning in the mouse over multiple generations. *Cell Stem Cell*.

[24] Gao R., Wang C., Gao Y. (2018). Inhibition of aberrant DNA re-methylation improves post-implantation development of somatic cell nuclear transfer embryos. *Cell stem cell*.

[25] Matoba S., Wang H., Jiang L. (2018). Loss of H3K27me3 imprinting in somatic cell nuclear transfer embryos disrupts post-implantation development. *Cell stem cell*.

[26] Chen M., Zhu Q., Li C. (2020). Chromatin architecture reorganization in murine somatic cell nuclear transfer embryos. *Nature Communications*.

[27] Inoue K., Kohda T., Sugimoto M. (2010). Impeding Xist expression from the active X chromosome improves mouse somatic cell nuclear transfer. *Science*.

[28] Chan M. M., Smith Z. D., Egli D., Regev A., Meissner A. (2012). Mouse ooplasm confers context-specific reprogramming capacity. *Nature Genetics*.

[29] Lu F., Liu Y., Inoue A., Suzuki T., Zhao K., Zhang Y. (2016). Establishing chromatin regulatory landscape during mouse preimplantation development. *Cell*.

[30] Bird A. (2002). DNA methylation patterns and epigenetic memory. *Genes & Development*.

[31] Smith Z. D., Meissner A. (2013). DNA methylation: roles in mammalian development. *Nature Reviews. Genetics*.

[32] Okano M., Bell D. W., Haber D. A., Li E. (1999). DNA methyltransferases Dnmt3a and Dnmt3b are essential for de novo methylation and mammalian development. *Cell*.

[33] Wu H., Zhang Y. (2014). Reversing DNA methylation: mechanisms, genomics, and biological functions. *Cell*.

[34] Hermann A., Goyal R., Jeltsch A. (2004). The Dnmt1 DNA-(cytosine-C5)-methyltransferase Methylates DNA Processively with High Preference for Hemimethylated Target Sites. *The Journal of Biological Chemistry*.

[35] Wu X., Zhang Y. (2017). TET-mediated active DNA demethylation: mechanism, function and beyond. *Nature Reviews Genetics*.

[36] Verma N., Pan H., Doré L. C. (2018). TET proteins safeguard bivalent promoters from de novo methylation in human embryonic stem cells. *Nature Genetics*.

[37] Kohli R. M., Zhang Y. (2013). TET enzymes, TDG and the dynamics of DNA demethylation. *Nature*.

[38] Guo F., Li X., Liang D. (2014). Active and passive demethylation of male and female pronuclear DNA in the mammalian zygote. *Cell Stem Cell*.

[39] Shen L., Inoue A., He J., Liu Y., Lu F., Zhang Y. (2014). Tet3 and DNA replication mediate demethylation of both the maternal and paternal genomes in mouse zygotes. *Cell Stem Cell*.

[40] Li E., Bestor T. H., Jaenisch R. (1992). Targeted mutation of the DNA methyltransferase gene results in embryonic lethality. *Cell*.

[41] Kang J., Lienhard M., Pastor W. A. (2015). Simultaneous deletion of the methylcytosine oxidases Tet1 and Tet3 increases transcriptome variability in early embryogenesis. *Proceedings of the National Academy of Sciences of the United States of America*.

[42] Gu T.-P., Guo F., Yang H. (2011). The role of Tet3 DNA dioxygenase in epigenetic reprogramming by oocytes. *Nature*.

[43] Wossidlo M., Nakamura T., Lepikhov K. (2011). 5-Hydroxymethylcytosine in the mammalian zygote is linked with epigenetic reprogramming. *Nature Communications*.

[44] Dean W., Santos F., Stojkovic M. (2001). Conservation of methylation reprogramming in mammalian development: aberrant reprogramming in cloned embryos. *Proceedings of the National Academy of Sciences of the United States of America*.

[45] Peat J. R., Reik W. (2012). Incomplete methylation reprogramming in SCNT embryos. *Nature Genetics*.

[46] Martin C., Zhang Y. (2005). The diverse functions of histone lysine methylation. *Nature Reviews Molecular Cell Biology*.

[47] Grunstein M. (1997). Histone acetylation in chromatin structure and transcription. *Nature*.

[48] Tessarz P., Kouzarides T. (2014). Histone core modifications regulating nucleosome structure and dynamics. *Nature Reviews. Molecular Cell Biology*.

[49] Becker J. S., Nicetto D., Zaret K. S. (2016). H3K9me3-dependent heterochromatin: barrier to cell fate changes. *Trends in Genetics*.

[50] Liu X., Wang Y., Gao Y. (2018). H3K9 demethylase KDM4E is an epigenetic regulator for bovine embryonic development and a defective factor for nuclear reprogramming. *Development*.

[51] Ruan D., Peng J., Wang X. (2018). XIST derepression in active X chromosome hinders pig somatic cell nuclear transfer. *Stem Cell Reports*.

[52] Di Croce L., Helin K. (2013). Transcriptional regulation by polycomb group proteins. *Nature Structural & Molecular Biology*.

[53] Simon J. A., Kingston R. E. (2009). Mechanisms of polycomb gene silencing: knowns and unknowns. *Nature Reviews Molecular Cell Biology*.

[54] Liu X., Wang C., Liu W. (2016). Distinct features of H3K4me3 and H3K27me3 chromatin domains in pre-implantation embryos. *Nature*.

[55] Zheng H., Huang B., Zhang B. (2016). Resetting epigenetic memory by reprogramming of histone modifications in mammals. *Molecular Cell*.

[56] Inoue A., Chen Z., Yin Q., Zhang Y. (2018). Maternal Eed knockout causes loss of H3K27me3 imprinting and random X inactivation in the extraembryonic cells. *Genes & Development*.

[57] Zhang W., Chen Z., Yin Q., Zhang D., Racowsky C., Zhang Y. (2019). Maternal-biased H3K27me3 correlates with paternal-specific gene expression in the human morula. *Genes & Development*.

[58] Xu Q., Xie W. (2018). Epigenome in early mammalian development: inheritance, reprogramming and establishment. *Trends in Cell Biology*.

[59] Xu R., Li C., Liu X., Gao S. (2020). Insights into epigenetic patterns in mammalian early embryos. *Protein & cell*.

[60] Xie B., Zhang H., Wei R. (2016). Histone H3 lysine 27 trimethylation acts as an epigenetic barrier in porcine nuclear reprogramming. *Reproduction*.

[61] Zhou C., Wang Y., Zhang J. (2018). H3K27me3 is an epigenetic barrier while KDM6A overexpression improves nuclear reprogramming efficiency. *FASEB journal*.

[62] Okae H., Matoba S., Nagashima T. (2014). RNA sequencing-based identification of aberrant imprinting in cloned mice. *Human Molecular Genetics*.

[63] Itoh M., Yoshida Y., Nishida K., Narimatsu M., Hibi M., Hirano T. (2000). Role of Gab1 in heart, placenta, and skin development and growth factor- and cytokine-induced extracellular signal-regulated kinase mitogen-activated protein kinase activation. *Molecular and Cellular Biology*.

[64] Miri K., Latham K., Panning B., Zhong Z., Andersen A., Varmuza S. (2013). The imprinted polycomb group gene Sfmbt2 is required for trophoblast maintenance and placenta development. *Development*.

[65] Inoue A., Jiang L., Lu F., Suzuki T., Zhang Y. (2017). Maternal H3K27me3 controls DNA methylation-independent imprinting. *Nature*.

[66] Inoue K., Ogonuki N., Kamimura S. (2020). Loss of H3K27me3 imprinting in the Sfmbt2 miRNA cluster causes enlargement of cloned mouse placentas. *Nature Communications*.

[67] Wang L. Y., Li Z. K., Wang L. B. (2020). Overcoming intrinsic H3K27me3 imprinting barriers improves post-implantation development after somatic cell nuclear transfer. *Cell stem cell*.

[68] Yang L., Song L., Liu X., Bai L., Li G. (2018). KDM6A and KDM6B play contrasting roles in nuclear transfer embryos revealed by MERVL reporter system. *EMBO reports*.

[69] Hanna C. W., Pérez-Palacios R., Gahurova L. (2019). Endogenous retroviral insertions drive non-canonical imprinting in extra-embryonic tissues. *Genome Biology*.

[70] Zhang B., Zheng H., Huang B. (2016). Allelic reprogramming of the histone modification H3K4me3 in early mammalian development. *Nature*.

[71] Dahl J. A., Jung I., Aanes H. (2016). Broad histone H3K4me3 domains in mouse oocytes modulate maternal-to-zygotic transition. *Nature*.

[72] Hörmanseder E., Simeone A., Allen G. E. (2017). H3K4 methylation-dependent memory of somatic cell identity inhibits reprogramming and development of nuclear transfer embryos. *Cell stem cell*.

[73] Zhou C., Zhang J., Zhang M. (2020). Transcriptional memory inherited from donor cells is a developmental defect of bovine cloned embryos. *FASEB journal*.

[74] Kurdistani S. K., Tavazoie S., Grunstein M. (2004). Mapping global histone acetylation patterns to gene expression. *Cell*.

[75] Wu J., Xu J., Liu B. (2018). Chromatin analysis in human early development reveals epigenetic transition during ZGA. *Nature*.

[76] Zhang B., Wu X., Zhang W. (2018). Widespread enhancer dememorization and promoter priming during parental-to-zygotic transition. *Molecular cell*.

[77] Wang F., Kou Z., Zhang Y., Gao S. (2007). Dynamic reprogramming of histone acetylation and methylation in the first cell cycle of cloned mouse embryos. *Biology of Reproduction*.

[78] Enright B. P., Kubota C., Yang X., Tian X. C. (2003). Epigenetic characteristics and development of embryos cloned from donor cells treated by trichostatin A or 5-aza-2'-deoxycytidine. *Biology of Reproduction*.

[79] Yang G., Zhang L., Liu W. (2020). Dux-mediated correactions of aberrant H3K9ac during 2-cell genome activation optimize efficiency of somatic cell nuclear transfer. *Cell stem cell*.

[80] Van Thuan N., Bui H. T., Kim J. H. (2009). The histone deacetylase inhibitor scriptaid enhances nascent mRNA production and rescues full-term development in cloned inbred mice. *Reproduction*.

[81] Tsuji Y., Kato Y., Tsunoda Y. (2009). The developmental potential of mouse somatic cell nuclear-transferred oocytes treated with trichostatin A and 5-aza-2'-deoxycytidine. *Zygote*.

[82] Yang P., Wu W., Macfarlan T. S. (2015). Maternal histone variants and their chaperones promote paternal genome activation and boost somatic cell reprogramming. *BioEssays: News and Reviews in Molecular, Cellular and Developmental Biology*.

[83] Talbert P. B., Henikoff S. (2017). Histone variants on the move: substrates for chromatin dynamics. *Nature Reviews Molecular Cell Biology*.

[84] Filipescu D., Szenker E., Almouzni G. (2013). Developmental roles of histone H3 variants and their chaperones. *Trends in Genetics*.

[85] Wen D., Banaszynski L. A., Rosenwaks Z., Allis C. D., Rafii S. (2014). H3.3 replacement facilitates epigenetic reprogramming of donor nuclei in somatic cell nuclear transfer embryos. *Nucleus*.

[86] Kong Q., Banaszynski L. A., Geng F. (2018). Histone variant H3.3-mediated chromatin remodeling is essential for paternal genome activation in mouse preimplantation embryos. *The Journal of Biological Chemistry*.

[87] Nashun B., Akiyama T., Suzuki M. G., Aoki F. (2011). Dramatic replacement of histone variants during genome remodeling in nuclear-transferred embryos. *Epigenetics*.

[88] Chang C. C., Gao S., Sung L. Y. (2010). Rapid elimination of the histone variant MacroH2A from somatic cell heterochromatin after nuclear transfer. *Cellular Reprogramming*.

[89] Gao S., Chung Y. G., Parseghian M. H., King G. J., Adashi E. Y., Latham K. E. (2004). Rapid H1 linker histone transitions following fertilization or somatic cell nuclear transfer: evidence for a uniform developmental program in mice. *Developmental Biology*.

[90] Teranishi T., Tanaka M., Kimoto S. (2004). Rapid replacement of somatic linker histones with the oocyte-specific linker histone H1foo in nuclear transfer. *Developmental Biology*.

[91] Sahakyan A., Yang Y., Plath K. (2018). The role of Xist in X-chromosome dosage compensation. *Trends in Cell Biology*.

[92] Plath K., Fang J., Mlynarczyk-Evans S. K. (2003). Role of histone H3 lysine 27 methylation in X inactivation. *Science*.

[93] Cao R., Wang L., Wang H. (2002). Role of histone H3 lysine 27 methylation in polycomb-group silencing. *Science*.

[94] Lee J. T., Bartolomei M. S. (2013). X-inactivation, imprinting, and long noncoding RNAs in health and disease. *Cell*.

[95] Ohhata T., Wutz A. (2013). Reactivation of the inactive X chromosome in development and reprogramming. *Cellular and Molecular Life Sciences: CMLS*.

[96] Xue F., Tian X. C., Du F. (2002). Aberrant patterns of X chromosome inactivation in bovine clones. *Nature Genetics*.

[97] Jiang L., Lai L., Samuel M., Prather R. S., Yang X., Tian X. C. (2008). Expression of X-linked genes in deceased neonates and surviving cloned female piglets. *Molecular Reproduction and Development*.

[98] Matoba S., Inoue K., Kohda T. (2011). RNAi-mediated knockdown of Xist can rescue the impaired postimplantation development of cloned mouse embryos. *Proceedings of the National Academy of Sciences of the United States of America*.

[99] Gao L., Wu K., Liu Z. (2018). Chromatin accessibility landscape in human early embryos and its association with evolution. *Cell*.

[100] Wu J., Huang B., Chen H. (2016). The landscape of accessible chromatin in mammalian preimplantation embryos. *Nature*.

[101] Jachowicz J. W., Bing X., Pontabry J., Bošković A., Rando O. J., Torres-Padilla M. E. (2017). LINE-1 activation after fertilization regulates global chromatin accessibility in the early mouse embryo. *Nature Genetics*.

[102] Miyamoto K., Nguyen K. T., Allen G. E. (2018). Chromatin accessibility impacts transcriptional reprogramming in oocytes. *Cell Reports*.

[103] Djekidel M. N., Inoue A., Matoba S. (2018). Reprogramming of chromatin accessibility in somatic cell nuclear transfer is DNA replication independent. *Cell Reports*.

[104] Fullwood M. J., Liu M. H., Pan Y. F. (2009). An oestrogen-receptor-*α*-bound human chromatin interactome. *Nature*.

[105] Atlasi Y., Stunnenberg H. G. (2017). The interplay of epigenetic marks during stem cell differentiation and development. *Nature Reviews. Genetics*.

[106] Ke Y., Xu Y., Chen X. (2017). 3D chromatin structures of mature gametes and structural reprogramming during mammalian embryogenesis. *Cell*.

[107] Du Z., Zheng H., Huang B. (2017). Allelic reprogramming of 3D chromatin architecture during early mammalian development. *Nature*.

[108] Chen X., Ke Y., Wu K. (2019). Key role for CTCF in establishing chromatin structure in human embryos. *Nature*.

[109] Brambrink T., Hochedlinger K., Bell G., Jaenisch R. (2006). ES cells derived from cloned and fertilized blastocysts are transcriptionally and functionally indistinguishable. *Proceedings of the National Academy of Sciences of the United States of America*.

[110] Egli D., Rosains J., Birkhoff G., Eggan K. (2007). Developmental reprogramming after chromosome transfer into mitotic mouse zygotes. *Nature*.

[111] Campbell K. H., Loi P., Otaegui P. J., Wilmut I. (1996). Cell cycle co-ordination in embryo cloning by nuclear transfer. *Reviews of Reproduction*.

[112] Prather R. S., Kühholzer B., Lai L., Park K. W. (2000). Changes in the structure of nuclei after transfer to oocytes. *Cloning*.

[113] Cibelli J. B., Stice S. L., Golueke P. J. (1998). Cloned transgenic calves produced from nonquiescent fetal fibroblasts. *Science*.

[114] Kato Y., Tani T., Sotomaru Y. (1998). Eight calves cloned from somatic cells of a single adult. *Science*.

[115] Wakayama T., Perry A. C., Zuccotti M., Johnson K. R., Yanagimachi R. (1998). Full-term development of mice from enucleated oocytes injected with cumulus cell nuclei. *Nature*.

[116] Baguisi A., Behboodi E., Melican D. T. (1999). Production of goats by somatic cell nuclear transfer. *Nature Biotechnology*.

[117] Onishi A., Iwamoto M., Akita T. (2000). Pig cloning by microinjection of fetal fibroblast nuclei. *Science*.

[118] Polejaeva I. A., Chen S. H., Vaught T. D. (2000). Cloned pigs produced by nuclear transfer from adult somatic cells. *Nature*.

[119] Chesné P., Adenot P. G., Viglietta C., Baratte M., Boulanger L., Renard J. P. (2002). Cloned rabbits produced by nuclear transfer from adult somatic cells. *Nature Biotechnology*.

[120] Shin T., Kraemer D., Pryor J. (2002). A cat cloned by nuclear transplantation. *Nature*.

[121] Woods G. L., White K. L., Vanderwall D. K. (2003). A mule cloned from fetal cells by nuclear transfer. *Science*.

[122] Galli C., Lagutina I., Crotti G. (2003). A cloned horse born to its dam twin. *Nature*.

[123] Zhou Q., Renard J. P., Le Friec G. (2003). Generation of fertile cloned rats by regulating oocyte activation. *Science*.

[124] Lee B. C., Kim M. K., Jang G. (2005). Dogs cloned from adult somatic cells. *Nature*.

[125] Li Z., Sun X., Chen J. (2006). Cloned ferrets produced by somatic cell nuclear transfer. *Developmental Biology*.

[126] Shi D., Lu F., Wei Y. (2007). Buffalos (Bubalus bubalis) cloned by nuclear transfer of somatic cells. *Biology of Reproduction*.

[127] Wani N. A., Wernery U., Hassan F. A., Wernery R., Skidmore J. A. (2010). Production of the first cloned camel by somatic cell nuclear Transfer1. *Biology of Reproduction*.

